# Incidence, risk factors and outcome of TRALI after cardiac surgery

**DOI:** 10.1186/cc12311

**Published:** 2013-03-19

**Authors:** A Dijkhuizen, R De Bruin, S Arbous

**Affiliations:** 1 Leiden University Medical Center, Leiden, the Netherlands

## Introduction

Transfusion-related acute lung injury (TRALI) is a syndrome that presents as a sudden onset of respiratory distress 6 hours after transfusion of blood products. The diagnosis is based on clinical and radiographic findings. Particularly at risk for TRALI are cardiac surgery patients. However, specific patient risk factors and data on outcome are largely unknown. The aim of this study was to investigate incidence, risk factors and outcome of TRALI in cardiac surgical patients on cardiopulmonary bypass.

## Methods

All thoracic surgery patients from a university hospital in the Netherlands of 18 years and older admitted to the ICU from January 2009 until December 2011 were screened. Included patients were observed during surgery and the first 24 hours on the ICU for the onset of possible TRALI. The Canadian Consensus Conference TRALI definition was used. Two independent physicians blinded to the predictor variables scored the chest radiographs for the onset of bilateral interstitial abnormalities on 2K monitors. When interpretation differed, chest radiographs were reviewed by a third physician to achieve consensus. The European System for Cardiac Operative Risk Evaluation (EURO score) and the American Association of Anesthesiology (ASA) were scored before surgery. By calculating the Acute Physiology and Chronic Health Evaluation (APACHE) II and IV scores the severity of illness was determined on arrival in the ICU.

## Results

In total, 1,787 cardiac surgical patients were included. A total of 19 (1.1%) patients developed TRALI within 24 hours following surgery. Patients developing TRALI were older compared with patients not developing TRALI, mean age respectively 71 and 65 years (*P *= 0.035). Furthermore, patients developing TRALI had higher APACHE II, APACHE IV, EURO and ASA score (*P *= 0.000, *P *= 0.000, *P *= 0.000 and *P *= 0.37). Compared with patients not developing TRALI, patients developing TRALI had a longer cardiopulmonary bypass time. Of the 19 patients developing TRALI, four patients (21.1%) died during ICU stay, in total six patients (31.6%) died during hospital stay. Patients developing TRALI were ventilated longer and had a longer ICU and hospital stay.

## Conclusion

Older age, higher APACHE II, IV, EURO and ASA scores and a longer time on cardiopulmonary bypass and ventilation time are risk factors for the development of TRALI. The ICU and hospital stay are longer, and the prognosis is bad for patients developing TRALI.
